# Supplementary material for: Multiplicity of type 6 secretion system toxins limits the evolution of resistance
Source: Proc Natl Acad Sci U S A. 2025 Jan 9;122(2):e2416700122. doi: 10.1073/pnas.2416700122 (PMC11745330; doi:10.1073/pnas.2416700122)
Supplement: Supplementary file 1 — Appendix 01 (PDF) [file pnas.2416700122.sapp.pdf]

Supplementary Information

Multiplicity of Type 6 Secretion System toxins limits the evolution of resistance

William P. J. Smith*[1], Ewan Armstrong-Bond[1], Katharine Z. Coyte[1], Christopher G. Knight[2], Marek Basler[3], Michael A. Brockhurst*[1]

*Corresponding authors:
william.smith-4@manchester.ac.uk,
michael.brockhurst@manchester.ac.uk

Affiliations:

1. Division of Evolution, Infection and Genomics, Faculty of Biology, Medicine and Health, University of Manchester, United Kingdom;
2. Department of Earth and Environmental Sciences, Faculty of Science and Engineering, University of Manchester, United Kingdom;
3. Biozentrum, University of Basel, Switzerland.

Supplementary Tables

Table S1: Agent-based model variables

**Variable for cell *i*:**	**Symbol**	**Units**
Position vector	$\mathbf{p}_i = (p_x, p_y, p_z)_i$	μm
Orientation unit vector	$\mathbf{v}_i = (v_x, v_y, v_z)_i$	-
Segment length	L_i	μm
Volume	$V_i = 4\pi R^3/3 + \pi L_i R^2$	μm^3
Intracellular toxin concentrations	$\mathbf{x} = (x_A, x_B)$	$cell^{-1}$
Cell integrity	$I = 1 - a_A x_A - a_B x_B - B_{AA} x_A^2 - B_{BB} x_B^2 - B_{AB} x_A x_B$	-

Table S2: Agent-based model parameters

Type	Parameter	Symbol	Value(s)	Units	Source
**Cells**	Cell radius	R	0.5	μm	Rudge2011
	Cell volume at birth	V_0	0.54	μm^3	Smith2017
	Max cell growth rate	k_{max}	1.0–0.3	h^{-1}	Rudge2011
	Random noise in division volume	η_{div}	0.027	%	Smith2017
	Cell division orientation noise	η_{orient}	0.2	%	Smith2017
**T6SS attacks**	Attacker firing rate	k_{fire}	5.0	$cell^{-1}\ h^{-1}$	Smith2020
	Dose of effector i translocated per hit	d_i	0-1	units	This study
	Extracellular needle length	L_{needle}	0.5	μm	Smith2020
	Min. needle penetration for hit	$L_{penetration}$	0.01	μm	Smith2020
	Weapon cost per unit secretion	c	0.0	h	This study
**T6SS response**	Sensitivity to toxin i	a_i	0.0–1.0	-	This study
	Coefficient of interaction between toxins i and j	B_{ij}	−0.3–0.3	-	This study
	Lysis delay following lethal dose (l=0)	k_{lysis}	0.125	h	Smith2020
**Numerical**	Simulation timestep	Δt	0.05	h	Rudge2011
	Cell / needle sorting grid size	h	10	μm	Smith2020
	Conjugate gradient absolute tolerance	e_{CG}	0.001	-	Rudge2011
	Max. contact iterations	Max_{iter}	8	-	Rudge2011
	Growth restriction factor	γ	500	-	Smith2017

Table S3: Bacterial strains used in this study

Strain	Genotype	Description	Purpose	Source
***A. baylyi* ADP1 attackers:**				
"*Ab*WT"	*rpsL-K88R, vipA-sfGFP clpV-mCherry2*	Parent ADP1 strain	-	Ringel2017 DH022
"*Ab*Tae1"	*rpsL-K88R vipA-sfGFP clpV-mCherry2 ΔACIAD0053 ΔACIAD1790 ΔACIAD3114 ΔACIAD3425*	ADP1 expressing only effector Tae1 (ACIAD0168)	T6SS Attacker armed with amidase toxin	Ringel2017 PR353
"*Ab*Tle1"	*rpsL-K88R vipA-sfGFP clpV-mCherry2 ΔACIAD0053 ΔACIAD0168 ΔACIAD1790 ΔACIAD3114*	ADP1 expressing only effector Tle1 (ACIAD3425)	T6SS Attacker armed with lipase toxin	Ringel2017 PR305
"*Ab*Tae1Tle1"	*rpsL-K88R vipA-sfGFP clpV-mCherry2 ΔACIAD0053 ΔACIAD1790 ΔACIAD3114*	ADP1 expressing both Tae1 and Tle1 effectors (ACIAD0168, ACIAD3425)	T6SS Attacker armed with both amidase and lipase toxins	Ringel2017 PR317
"*AbΔhcp*"	*rpsL-K88R, Δhcp vipA-sfGFP clpV-mCherry2*	ADP1 with no functional T6SS	T6SS-negative control to test EC adaptation to co-culture	Ringel2017 DH039
***E. coli* MG1655 defenders:**				
"AncG"	MG1655::eGFP Kan^R	Kan-resistant ancestral defender strain with eGFP tag	Experimental resistance evolution	Van Der Woude lab (University of York) via Michael J Bottery
"AncR"	MG1655::mCherry Kan^R	Kan-resistant ancestral defender strain with mCherry tag	Reference strain for cytometric fitness assay	Van Der Woude lab (University of York) via Michael J Bottery

Supplementary Figures

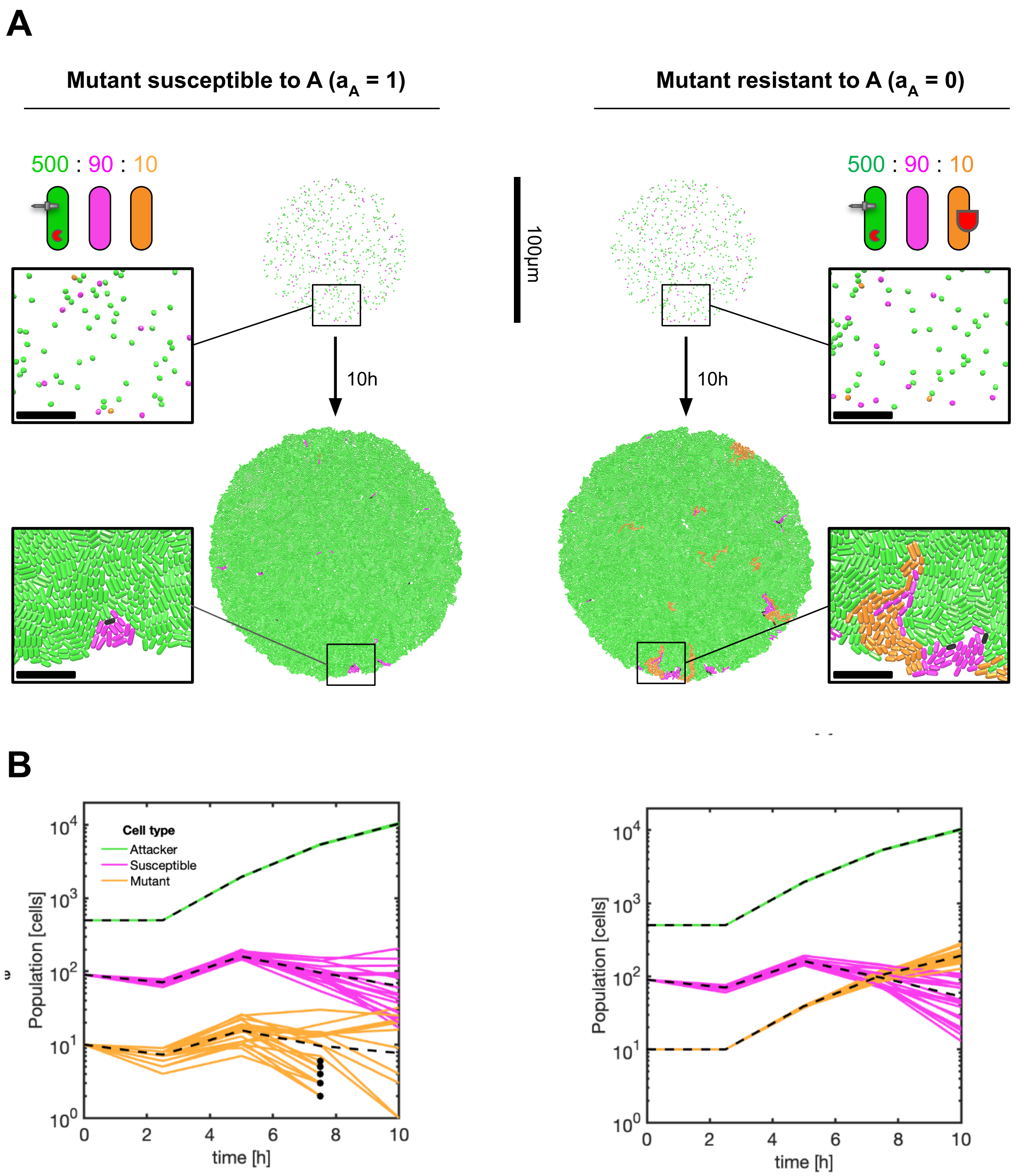

Fig. S1. Resistance selection simulations using an agent based model. To quantify evolutionary selection for T6SS resistant-strains, we simulate competitions between T6SS-armed attackers (green), susceptible cells (magenta), and rare mutant cells (orange) with variable sensitivities a_i to attackers' toxins. **(A)** Example competition simulations for mutants having high (a_A=1, left column) or low (a_A=0, right column) sensitivity to a focal toxin "A" (red pacmen). We initiate simulations by randomly scattering cells in a 500:90:10 (attacker:susceptible:mutant) ratio within a 100μm diameter circle (top row). Cells are then allowed to grow, divide and interact for 10h, before final frequencies of competing cells are assessed (bottom row). Scale bars: 100μm (whole colony images) and 10μm (zoomed sections). **(B)** Example population traces (coloured lines) for the scenario shown in A, indicating absence of selection for a T6SS-sensitive mutant (a_A=1, left column), but positive selection for a T6SS-resistance mutant (a_A=0, right column). Dashed black lines indicate means of N=20 simulation replicates; black circles mark events where the mutant population goes extinct due to T6SS killing.

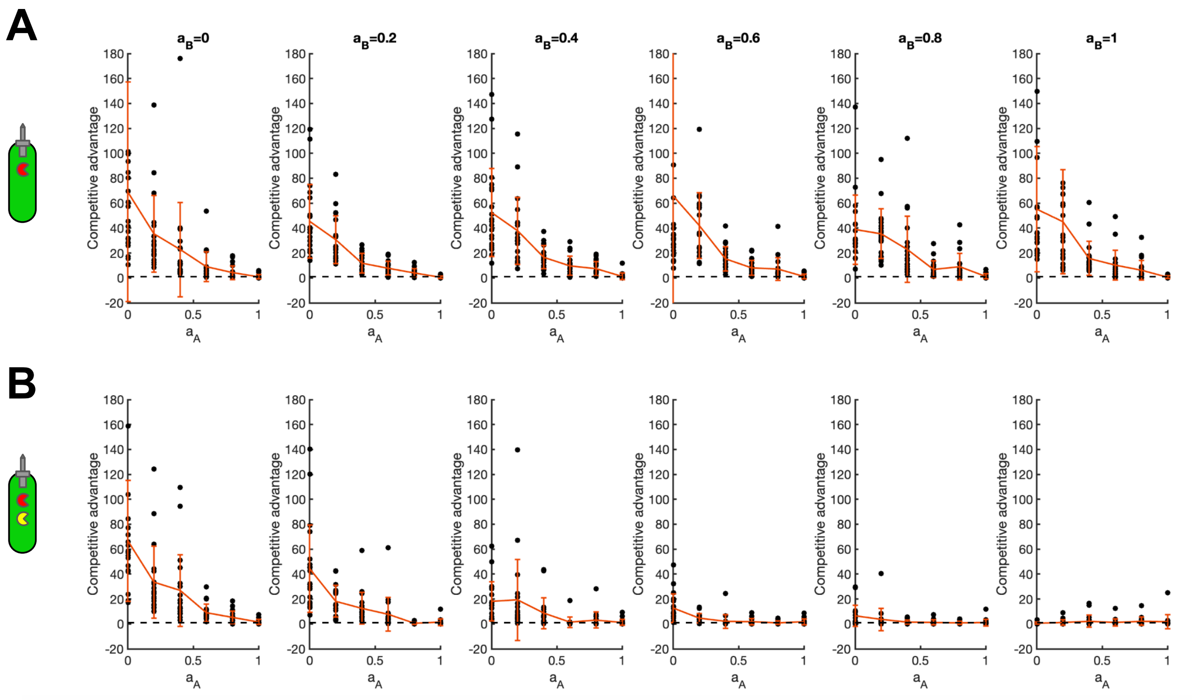

Figure S2: Multi-toxin attackers limit zone of positive selection for rare T6SS-resistant mutants. **(A)** Plots of Competitive advantage (without log transformation) chart selection for rare mutants, as a function of resistance to individual toxins (a_A, graph axes; a_B; columns), against attackers armed only with toxin “A” (red Pac-Man). Data replotted from Fig. 1D. **(B)** As (A) but against attackers armed with both “A” and “B” toxins (red and yellow pac-man). Data replotted from Fig. 1E. In A, B, black dots correspond to individual simulation replicates; red lines and bars show data means and standard deviations from N=20 0h replicate simulations, each initiated with random assortment of 500:90:10 (attacker:sensitive:resistant) cells. Dashed horizontal lines correspond to absence of selection (Competitive advantage = 1).

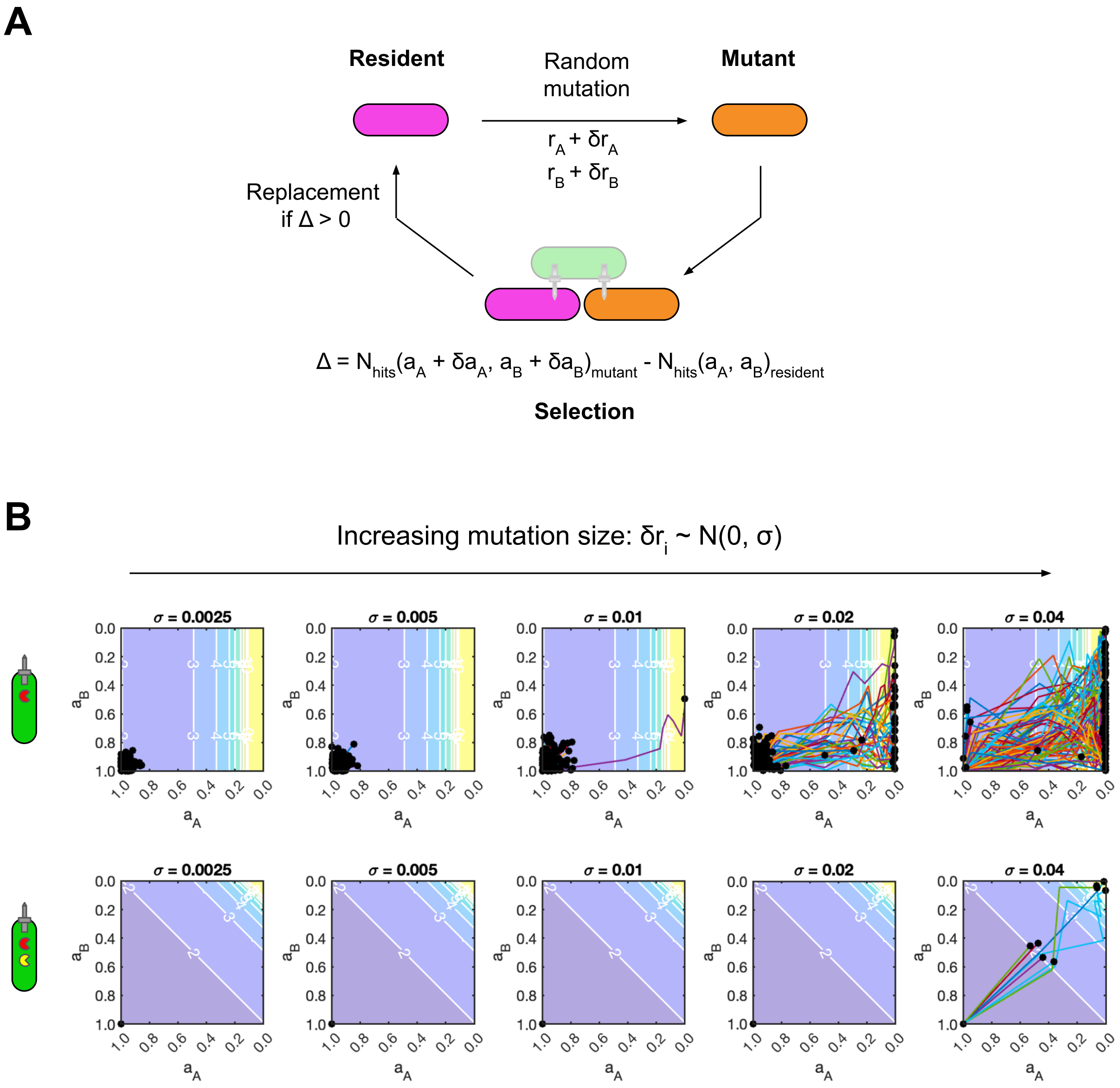

Fig. S3: Multi-toxin attackers supress resistance evolution at intermediate mutation rates with a simplified trajectory model. (A) simplified trajectory model replaces (computationally-intensive) agent-based simulations with a simple comparison of phenotypic resistance (N_{hits}) for the mutant and resident strain: if, for a given mutation $a_A + \delta a_A$, $a_B + \delta a_B$, N_{hits}(mutant) > N_{hits}(resident), the mutant replaces the resident. **(B)** Evolutionary trajectories (coloured lines) simulated using the simplified model, imposing selection by single-toxin (top row) or two-toxin attackers (bottom row), for increasing mutation step sizes σ (columns). Trajectories all share the starting point (a_A, a_B) = (1,1), and are plotted on a contour map showing phenotypic resistance $N_{hits}(a_A, a_B)$ for each coordinate (a_A, a_B) (see Methods). Trajectory endpoints are shown as black circles. N=100 trajectories per plot, each consisting of 100 sequential mutations.

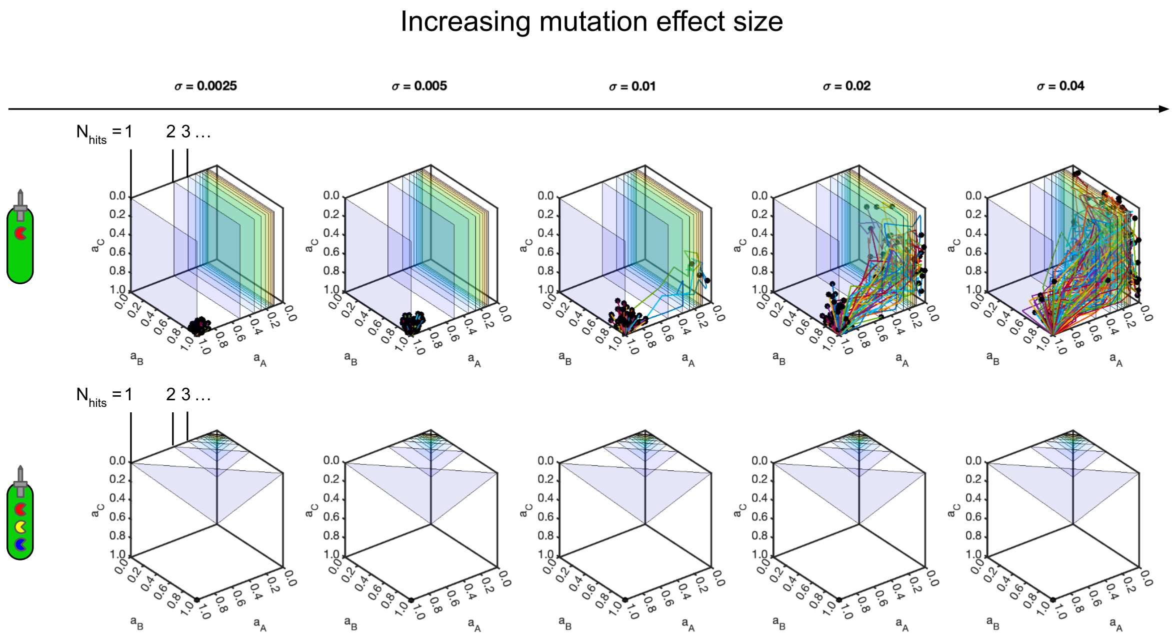

Fig. S4: Resistance suppression is enhanced by increased toxin arsenal size. Evolutionary trajectories (coloured lines) for simplified model incorporating three toxins (A, B and C) instead of two, comparing selection by single-toxin (top row) or three-toxin attackers (bottom row), for increasing mutation step sizes σ (columns). Trajectories all share the starting point (a_A, a_B, a_C) = (1,1,1). Coloured transparent surfaces show isoclines in phenotypic resistance (N_{hits} = 1,2,3…10) as annotated. Trajectory endpoints are shown as black circles. N=100 trajectories per plot, each consisting of 100 sequential mutations.

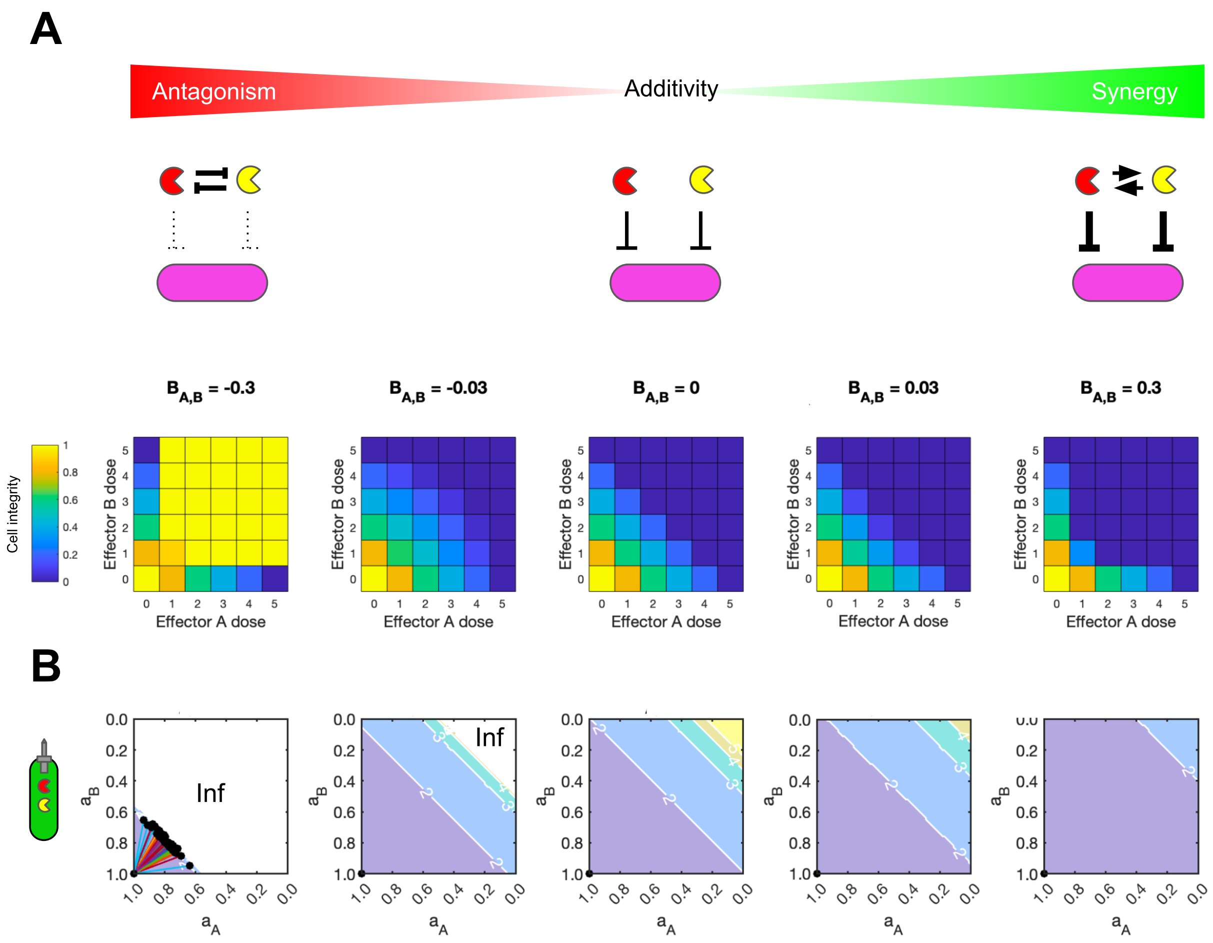

Fig. S5: Resistance suppression by multi-toxin attackers is enhanced by toxin synergy, but negated by toxin antagonism. (A) Using our simplified model, we consider the inclusion of antagonistic and synergistic interactions between two T6SS toxins (red and yellow Pac-Men), examining how these affect overall toxicity and the consequent potential for resistance evolution. Colormaps show residual cell integrity I of a focal cell (with $a_A = a_B = 0.2$) as a function of the dose of each effector, for increasing values of the effector interaction parameter $B_{A,B}$. For $B_{A,B} < 0$ (antagonism, left), the effectors are less toxic together than when secreted individually; for $B_{A,B} > 0$ (synergy, right) the reverse is true. **(B)** Evolutionary trajectories (coloured lines) simulated using the simplified model, imposing selection by two-toxin attackers for the $B_{1,2}$ values shown (columns). Trajectories all share the starting point $(a_A, a_B) = (1,1)$, and are plotted on a contour map showing phenotypic resistance $N_{hits}(a_A,a_B)$ for each coordinate (a_A,a_B). Note that in some cases antagonism becomes sufficiently strong that toxicity is completely abolished, such that $N_{hits} = \infty$ (Inf). Trajectory endpoints are shown as black circles. N=100 trajectories per plot, each consisting of 100 sequential mutations, mutation stepsize $\sigma = 0.1$.

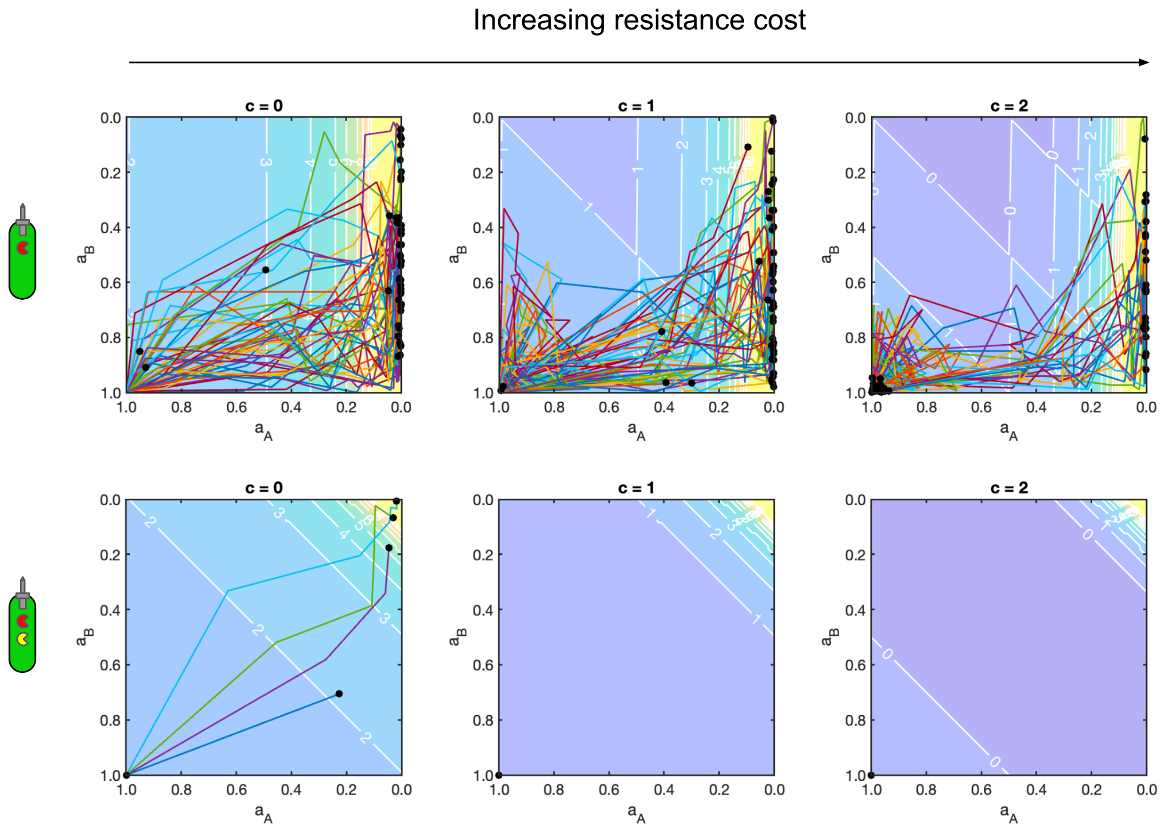

Fig. S6: Resistance suppression by multi-toxin attackers is enhanced by the addition of resistance fitness costs. Using our simplified model, we consider the inclusion of fitness costs that scale linearly with resistance to each effector. In this model, a resident or mutant strains' fitness ω is given by $\omega = N_{hits}(a_A, a_B) - c\ (a_A + a_B)$, with parameter c controlling the relative cost of resistance. Above, we show evolutionary trajectories (coloured lines) simulated using the simplified model, imposing selection by single-toxin (top row) or two-toxin attackers (bottom row), for increasing cost values c (columns). Trajectories all share the starting point $(a_A, a_B) = (1,1)$, and are plotted on a contour map showing phenotypic resistance $N_{hits}(a_A, a_B)$ for each coordinate (a_A, a_B). Trajectory endpoints are shown as black circles. N=100 trajectories per plot, each consisting of 100 sequential mutations.

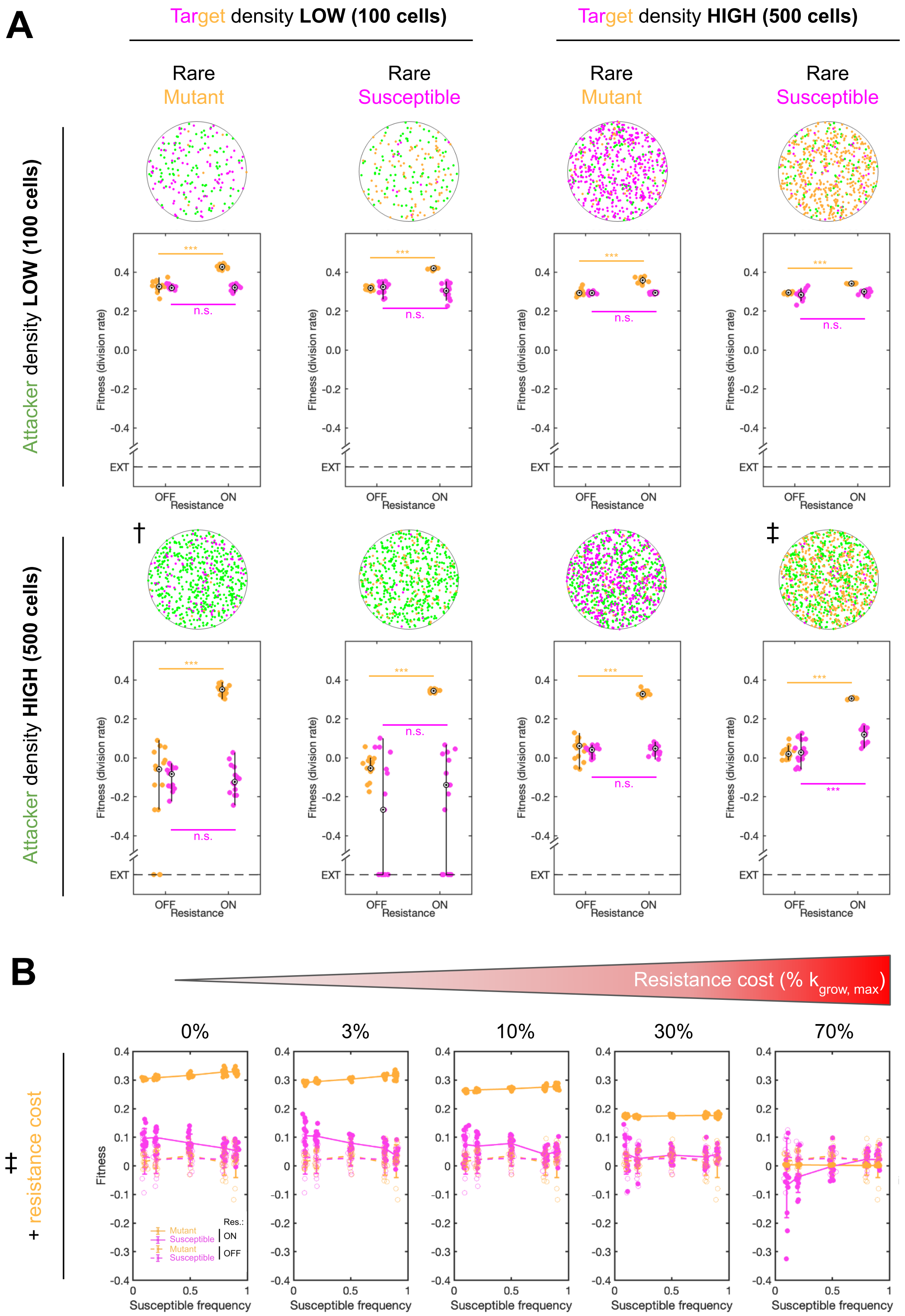

Fig. S7: Collective protection effects arising in agent-based model simulations. (A) Fitness (division rate) changes to susceptible (magenta) and mutant (orange) populations resulting from mutant resistance, tested for different attacker and target cell starting densities (LOW / HIGH: 100 and 500 cells) and mutant frequencies (Rare: frequency 0.1; examples shown in circles). Resistance OFF and ON correspond to mutant $a_A = 1.0$ and $a_A = 0.0$ respectively; black bars and circles show interquartile ranges and medians. Individual data points are shown as coloured circles; the horizontal dashed line corresponds to extinction (EXT; fitness = $-\infty$). Significance tests performed using Student's pairwise t-test; *** and n.s. denote $p<0.001$ and $p>0.05$. † indicates conditions used in Figs. 1 and 2; ‡ marks conditions with significant cross-protection. **(B)** Comparison of mutant and susceptible fitness for variable susceptible frequency and increasing mutant resistance costs. Individual data points are shown as coloured circles; lines and errorbars show data means and standard deviations (solid and dashed lines denote resistant and sensitive mutants, respectively). N=15 simulations each lasting 10h.

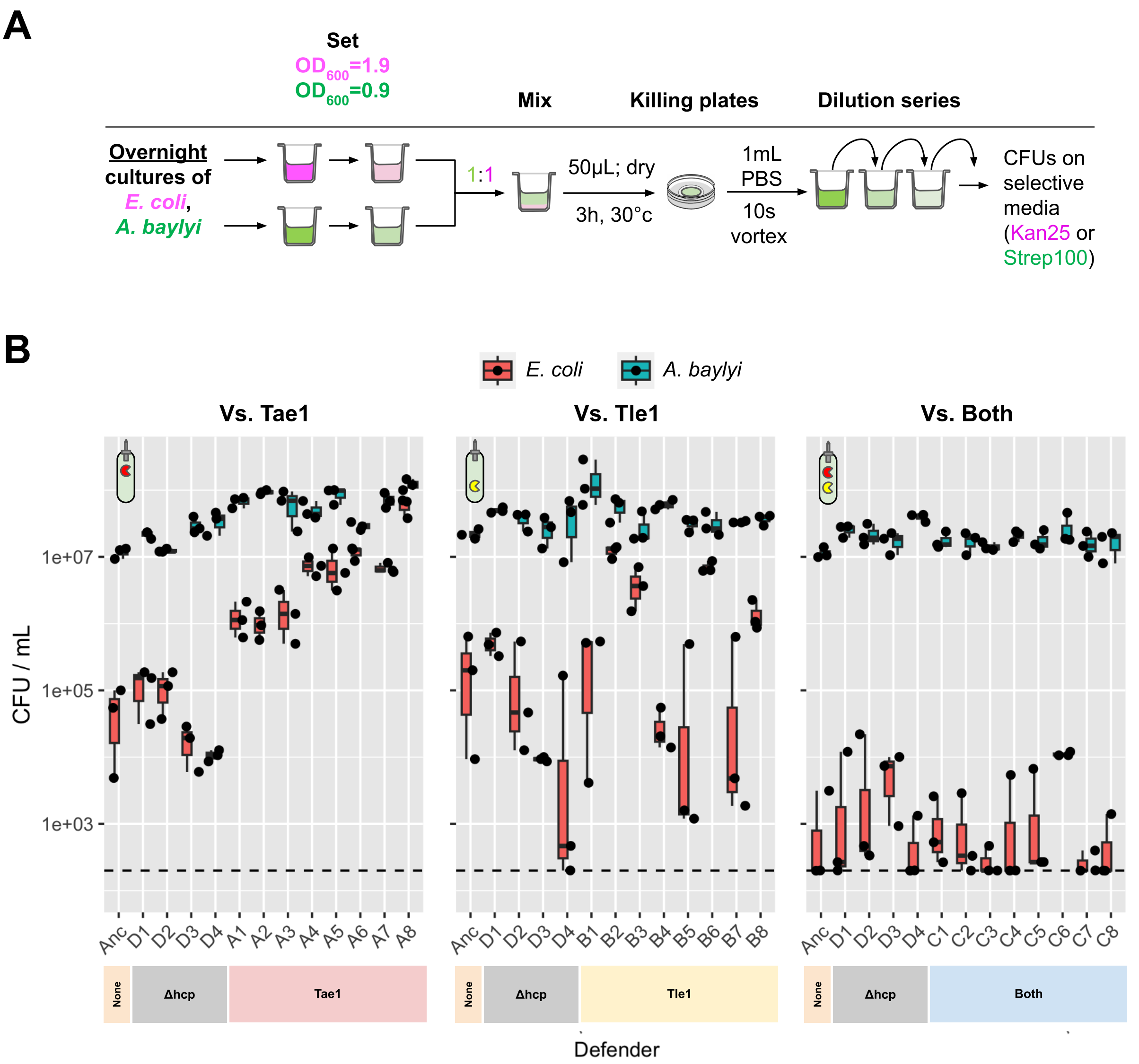

Fig. S8: Raw recovery data for end-point resistance assay. (A) Diagram summarising experimental workflow for end-point resistance assay, showing (from left to right) culture density normalisation, mixing, plating, recovery and CFU enumeration. **(B)** Raw CFU data comparing recovery of "Defender" *E. coli* end-point clones D1-4, A1-8, B1-8, C1-8 and the (Anc)estral strain (red), for co-culture with each of the three T6SS attackers (left, middle and right panels). These are data shown alongside *A. baylyi* recovery (teal), plotted as a control for condition uniformity. Box-and-whisker plots show data median, interquartile range and range; individual data points are plotted as black dots. Dashed black line shows the experiments' detection limit (200 CFU / mL). N=3 pseudobiological replicates; each data point represents the average of 3 technical plating replicates of the same dilution series. The coloured bars below each graph indicate the treatment against which that *E. coli* isolate evolved. *E. coli* recovery data shown are those shown in Figs. 3B (Ancestral strain only) and 3E.

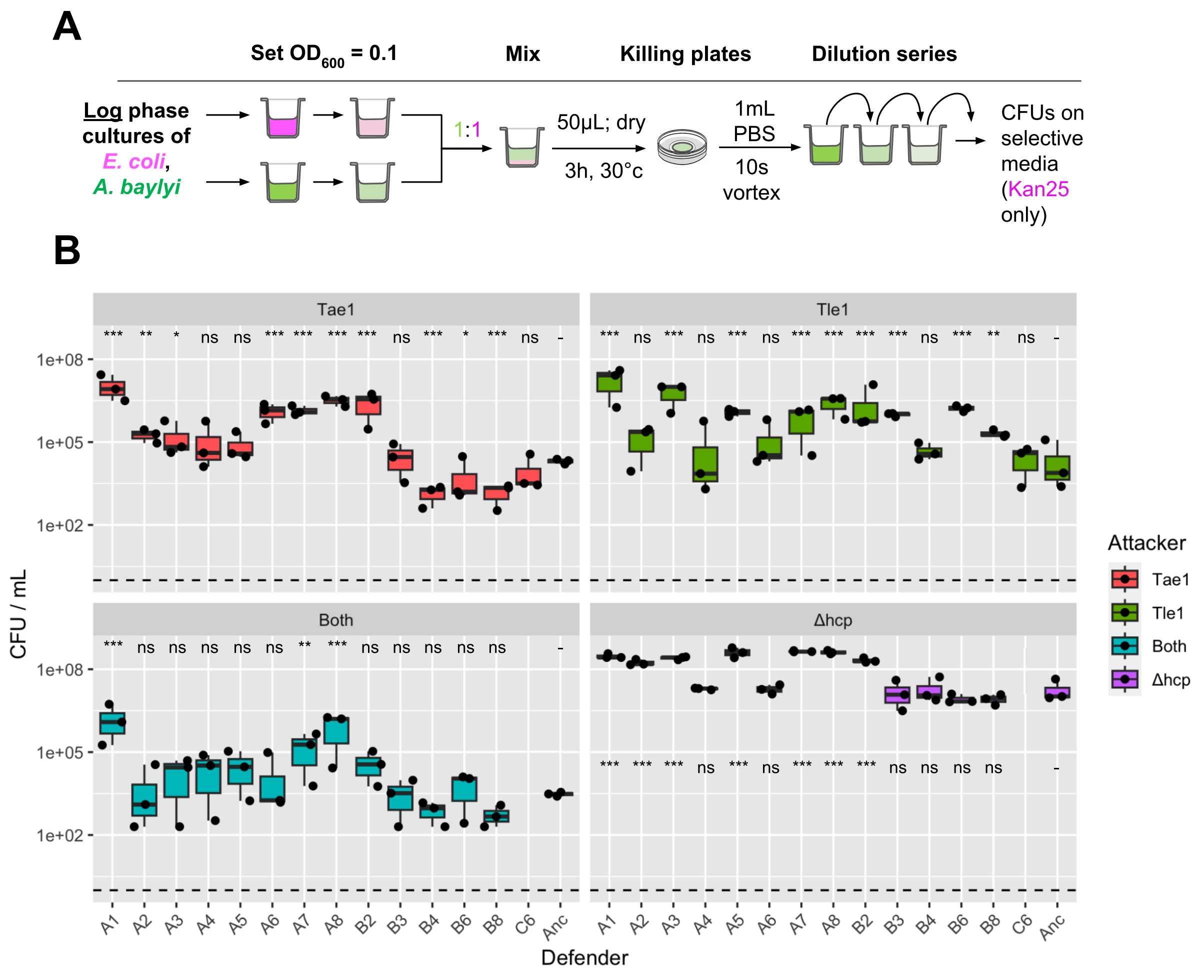

Fig. S9: Raw recovery data for cross-resistance assay. (A) Diagram summarising experimental workflow for cross resistance assay. **(B)** Raw CFU data comparing recovery of resistant *E. coli* end-point clones (A1-8, B2-4, B6, B8 and C6 and the (Anc)estral strain, when co-cultured with each of the four T6SS attackers (each shown as a separate panel). Box-and-whisker plots show data median, interquartile range and range; individual data points are plotted as black dots. Dashed black line shows the experiments' detection limit (200 CFU / mL). N=3 pseudobiological replicates; each data point represents the average of 3 technical plating replicates of the same dilution series. Data replotted from Fig. 4A and 4B. Note: isolate C6 was omitted from "Both" and "*Δhcp*" treatments. Significance codes: ***, **, *, ns respectively denote p<0.001, p<0.01, p<0.05 and p>0.05; individual isolates assessed for difference with ancestral strain using linear modelling (Dunnett's test for multiple comparisons).

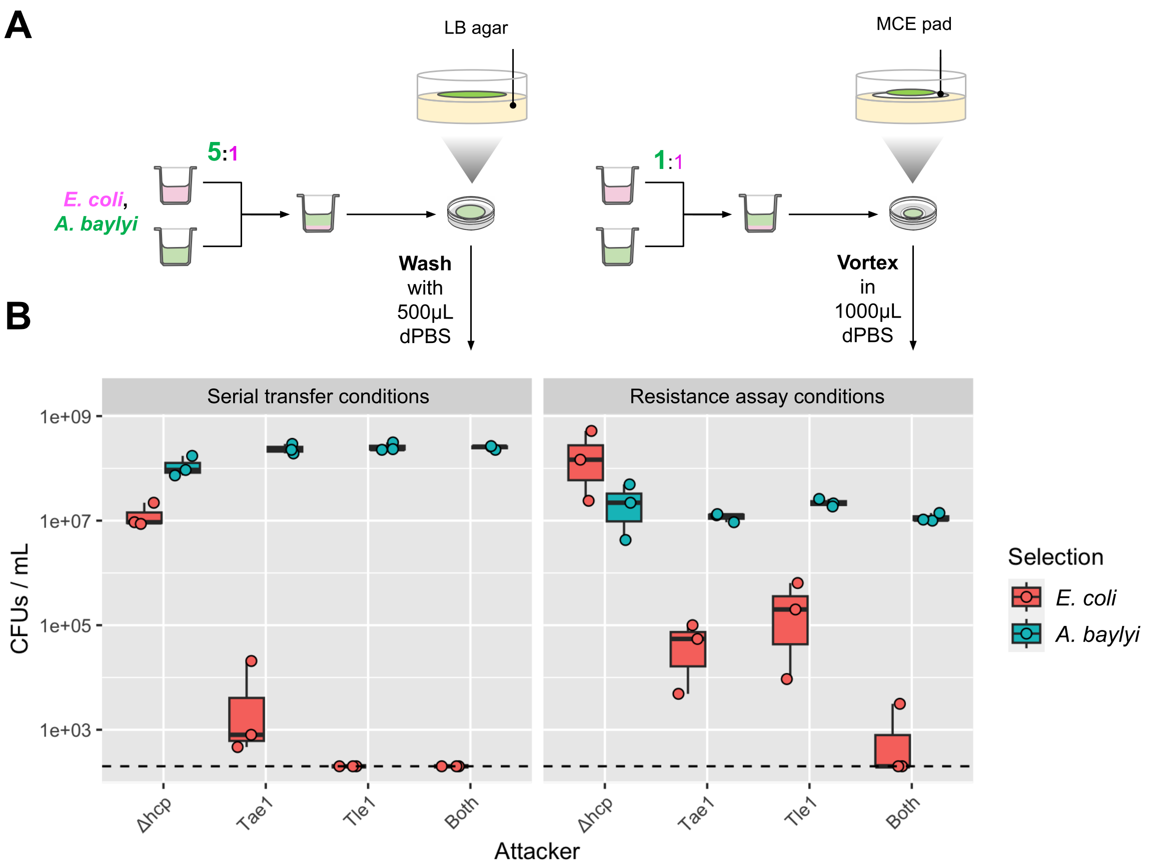

Fig. S10: Comparison of T6SS toxin lethality between serial transfer conditions and resistance assay conditions. (A) Protocol comparison between serial transfer experiments (left) and resistance assays (right), which differ in the initial ratio of *A. baylyi* : *E. coli* cells (5:1 or 1:1), the surface on which co-cultures are incubated (LB agar or Mixed [nitro]Cellulose Ester filter pads placed on LB agar), and the method used to recover cells (repeated washing or vortexing). **(B)** Raw CFU data comparing recovery of *E. coli* Ancestral strain (red) and *A. baylyi* attacker strains (teal) as a function of T6SS toxin treatment (x-axes) between these two experimental protocols. Dashed black line shows the experiments' detection limit (200 CFU / mL). N=3 pseudobiological replicates; each data point represents the average of 3 technical plating replicates of the same dilution series. Data shown under "Resistance assay conditions" are replotted from Fig. 3B.
